# A Redesigned Order Entry System for Reducing Low-Value Preprocedural Cardiology Consultations: Quality-Improvement Cohort Study

**DOI:** 10.2196/17669

**Published:** 2020-05-01

**Authors:** David E Winchester, Leigh Cagino

**Affiliations:** 1 Malcom Randall Veterans Affairs Medical Center Gainesville, FL United States

**Keywords:** quality improvement, preoperative care, medical order entry systems

## Abstract

**Background:**

Preprocedural cardiac evaluation is a common reason for outpatient cardiology visits. Many patients who are referred to cardiology clinics for preprocedural evaluation are at low risk of perioperative events and do not require any further management. Our facility treats patients over a large geographic area; avoiding low-value consultations reduces time and travel burdens for patients.

**Objective:**

Our study objective was to assess the impact of a novel algorithm in the electronic order entry system aimed to guide clinicians toward patients who may benefit from cardiovascular referral.

**Methods:**

We retrospectively reviewed in-person consultations and electronic consultations (e-consults) to our cardiology service before and after implementation of the novel algorithm to assess changes in patterns of care. Data were stored in a custom electronic database on internal servers.

**Results:**

We reviewed 603 consultations to our cardiology clinic and found that 89 (14.7%) were sent for preprocedural evaluation. Of these, 39 (43.8% of preprocedural consultations) were e-consults. After implementation, we reviewed 360 consultations. The proportion of consultations for preprocedural evaluation did not decrease (n=47, 13.0%; *P*=.39). We observed an absolute increase of 13.6% in the proportion of consultations ordered as e-consults (27/47, 57.4%). During the postintervention period, we received no remarks, concerns, or criticisms from ordering clinicians about the process change and no reports of adverse events.

**Conclusions:**

Implementation of an ordering algorithm to reduce low-value preprocedural cardiology evaluations did not lead to a reduction in the number of overall preprocedural cardiology consultations. The number of patients seen electronically increased, potentially improving clinic access and reducing travel burden for patients.

## Introduction

Preprocedural evaluation is a common reason for outpatient cardiology clinic referrals in both community and academic settings. Such referrals are sometimes made for patients undergoing minimal risk procedures with no history of and few risk factors for heart disease. Despite clear appropriate use criteria and guideline recommendations, unnecessary preprocedural testing is often performed, and preprocedural assessments infrequently result in modification of care [1].

For patients who plan to undergo elective procedures, the addition of a referral to a cardiologist for preprocedural cardiac evaluation may delay the procedure for days or weeks depending on clinic wait times. Due to regionalization of care within the US Veterans Health Administration (VHA) system, some patients are required to travel for hours each way to receive a specialty care referral.

In order to reduce low-yield preprocedural cardiac evaluation and minimize inconvenience to patients, we implemented an ordering algorithm to be used by all clinicians requesting cardiology consultation for preprocedural patients. We hypothesized that the algorithm would reduce the number of in-person clinic visits for preprocedural cardiac evaluation.

## Methods

**Figure 1:**
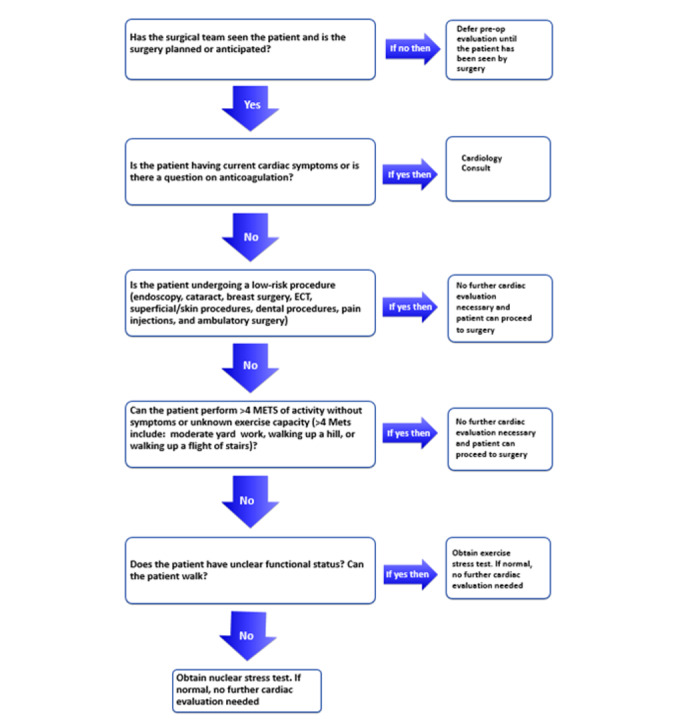
Flowchart of preprocedural consult guidance.

We conducted a quality-improvement project wherein we implemented a novel order entry system in our electronic health records at a single academically affiliated Veterans Affairs (VA) medical center. We devised a simple, stepwise algorithm to guide ordering clinicians on which patients need cardiology referral and which do not. The algorithm consisted of five questions assessing the patient’s cardiac symptoms, need for anticoagulation management, exercise capacity, procedural risk, and testing options (Figure 1). The intervention was built into the workflow of ordering a cardiology consultation, so that it could not be bypassed. All referring physicians were required to use the new algorithm, including those in surgery and subspecialties, anesthesia, primary care, and other procedural specialties. Prior to implementation, these clinicians were notified and educated about the algorithm via email correspondence.

Prior to our intervention, we analyzed the pattern of consultations received for the cardiology clinic over a 3-month period (June 1, 2015, to August 31, 2015). Variables evaluated included the proportion of cardiology consultations that were ordered for preprocedural assessment, the proportion of consultations ordered as e-consults versus in-person consultations, and the proportion of consultations that were converted from one type to another. As a balancing measure, after the intervention, we requested feedback via email from process stakeholders, including cardiology, primary care, and surgery clinicians. The e-consults at our facility consist of written and verbal asynchronous communication between the referring and consulted clinicians without any direct involvement of the patient. For cardiology at our facility, the same physicians provide both outpatient clinic and e-consult services.

Applying this algorithm to our baseline sample, we estimated that 50% of patients would not need clinic referrals if the algorithm was followed. We estimated that a review of consult requests over 6 weeks (approximately 300) would provide 80% power to detect a 50% reduction in preprocedural referrals with α of .05. The numbers of referrals before and after the intervention were compared by Fisher's exact test using SPSS version 25 (IBM Corporation).

In accordance with VA Handbook 1058.05, this project was performed with the purpose of improving quality of care and was determined to not qualify as human subject research. This manuscript was developed in accordance with the CONSORT-EHEALTH checklist [2].

## Results

A total of 963 consultations (603 before and 360 after the intervention) were evaluated. The overall proportions of cardiology referrals for preprocedural evaluation were similar in the before and after groups (n=89, 14.7% vs n=47, 13.0%; *P*=.39; odds ratio 0.87; 95% CI 0.59-1.27). Table 1 shows the changes in the distribution of how consultations were ordered and completed after the algorithm was introduced (2×4 Fisher exact test, *P*=.03). The proportion of consultations ordered as e-consults increased (n=39/89, 43.8% to n=27/47, 57.4%), while the proportion of patients seen in the clinic decreased (n=60/89, 67.4% to n=20/47, 42.6%). Feedback from cardiology and referring clinicians was positive. Cardiology clinicians reported that preprocedural referrals were often more complete (eg, patients were not referred without first seeing a surgeon or unless the consult included a specific question to address). The referring clinicians did not voice any concerns or criticisms about the new ordering algorithm.

**Table 1:** Distribution of preprocedural consultations before and after implementation of the order entry algorithm. The change in proportions was significant (*P*=.03).

Outcome	Before implementation (n=89), n (%)	After implementation (n=47), n (%)
Clinical consultation ordered and patient seen in clinic	49 (55.1)	17 (36.2)
Clinical consultation ordered and e-consult^a^ performed	1 (1.1)	3 (6.4)
E-consult ordered and patient seen in clinic	11 (12.4)	3 (6.4)
E-consult ordered and performed	28 (31.5)	24 (51.1)

^a^E-consult: electronic consultation.

## Discussion

### Principal Findings

Implementing an algorithm to reduce referrals for low-value preprocedural cardiac evaluation did not decrease the volume of referrals but did shift the ordering pattern to more e-consults. Based on an average volume of 20 preprocedural clinic visits per month, we estimate that 5 fewer patients per month were seen in the clinic because of the process change. Additionally, the burden on patients is reduced by eliminating travel for low-value care and reducing barriers to elective procedures.

The reasons why patients are commonly referred to cardiologists for preprocedural assessment are complex. The current American College of Cardiology/American Heart Association guidelines provide ample direction on how to adequately assess cardiovascular risk and suggest when further cardiac testing is indicated [1]. However, the guidelines do not specify which patients are likely to benefit from cardiologist expertise and which can be managed by primary care or anesthesiology alone. In 2003, Park et al [3] published a suggested strategy to determine which patients warranted specialty evaluation; their strategy was similar to the one we adopted for this project. Primary care scholarly literature, continuing medical education, and informal writings are replete with reviews on the topic of preprocedural assessment, demonstrating that this skill set is well within their purview [4,5]. The Centers for Medicare & Medicaid Services bundles preprocedural assessment with surgical reimbursement. This may lead to cardiology referrals where billing for separate evaluation and management services can be justified; however, these rules do not apply within VHA medical centers.

The preprocedural evaluation itself is of questionable clinical relevance. Among referring clinicians, there is a lack of consensus on what constitutes an appropriate consultation [6]. Referring clinicians commonly do not state a clear reason for cardiac evaluation and will use vague terminology such as “clear for surgery” [7]. In a recent study [8] of 273 referrals to cardiology for preprocedural evaluation, only 2% led to invasive intervention; 37% resulted in a medication change and 61% resulted in no changes or interventions following cardiology consultation. Kleinman et al [7] reviewed 202 preprocedural consultations and found that 52 (25.7%) had a change in preprocedural therapy. Most of these changes were related to uncontrolled hypertension or angina, which are both conditions that can be readily managed in a primary care setting. If we accept that preprocedural evaluation has limited clinical value, e-consults unfortunately do not directly address the root problem. In this context, e-consults function as a stopgap measure to reduce the burden of low-value care on facilities and patients; however, the burden on clinicians may not be substantially different. Adequately addressing the low value of preprocedural assessments will require, at a minimum, multidisciplinary agreement on which patients would benefit from them. After that, changes in front-line practice would require substantial effort, which may not provide a worthwhile return on investment of time and resources.

Adoption of the preprocedural assessment algorithm in our study did not reduce the number of referrals; however, we did see secondary evidence of improved clinic efficiency. Cardiology clinicians reported that when they saw patients in the clinic, the consultation referral was more often complete and included a specific question to address. Despite no decrease in overall referrals, more patients were evaluated using e-consults. Since their implementation in 2011, e-consults have been shown to be successful in VHA medical centers; both patients and clinicians were satisfied with the improvement in communication and timeliness of care [9]. Each time a patient was seen electronically instead of in person, the burden on the patient was also reduced. Approximately 43% of all veterans who receive VA care reside in nonurban areas where the average straight-line distance to the nearest VA health care facility is 23 miles [10]. Our facility is part of a network of 14 clinics and medical centers, only 3 of which offer outpatient cardiology care and where veterans may be required to drive up to 170 miles one way for an office visit.

Veteran patients are not substantially different from other populations; therefore, we do not believe that our intervention has any patient-specific limits on generalizability. It would be beneficial to study this algorithm outside the VA in an academic or private setting where e-consults are not widely used. Although the VA is not highly concerned with reimbursement from third-party payers, poor or inconsistent reimbursement for e-consults may limit adoption of similar practices in other care settings. There is potential for a decrease in overall use of preprocedural consultations, with benefits of decreased wait time and cost for patients.

We should note some limitations of our intervention. Formal tracking of clinical outcomes and downstream testing were beyond the scope of our research. As this is a report of a quality-improvement project, we are unable to provide some data that would be of interest, such as demographic information and medical history of the patients being evaluated. The postintervention sample (n=360) was higher than our projection of 300 because the quality improvement team divided the work into weeks of consultations and then collated the results of their reviews.

### Conclusions

Our intervention standardized the approach to ordering preprocedural cardiology referrals and enhanced the quality of communication in the referrals. Face-to-face consultations were reduced through use of e-consults, allowing veterans to avoid unnecessary and burdensome travel.

## Appendix

Multimedia Appendix 1 CONSORT-eHEALTH checklist (V 1.6.1).

## Abbreviations

e-consult: electronic consultation
VA: Veterans Affairs
VHA: Veterans Health Administration
